# Effects of weight loss after sleeve gastrectomy on left ventricular myocardial work in obese patients

**DOI:** 10.3389/fcvm.2025.1525746

**Published:** 2025-03-14

**Authors:** Xiao Ding, Xijun Zhang, Jingge Zhao, Changhua Wei, Shuaiwei Luo, Jianjun Yuan, Haohui Zhu

**Affiliations:** ^1^ Department of Ultrasonography, Henan Provincial People’s Hospital, Zhengzhou, Henan, China; ^2^ Department of Clinical Research Center, Henan Provincial People’s Hospital, Zhengzhou, Henan, China

**Keywords:** obesity, echocardiography, sleeve gastrectomy, left ventricular, myocardial work

## Abstract

**Background:**

Obesity is a global epidemic and a major risk factor for cardiovascular diseases. Laparoscopic sleeve gastrectomy (LSG) is an effective bariatric surgery, but its effect on cardiac functions remains unclear. This study aims to investigate the impact of weight loss after LSG on the left ventricular myocardial work (LVMW) in obese patients and explore the clinical value of the left ventricular pressure - strain loop (LV - PSL).

**Methods:**

Thirty - eight obese patients (body mass index ≥ 30 kg/m^2^) were enrolled preoperatively, and 31 patients completed the study after six months of follow - up. Clinical information, parameters from left ventricular myocardial work and traditional two - dimensional strain echocardiography were collected and analyzed.

**Results:**

After LSG, significant reductions in body mass index (BMI), diastolic blood pressure (DBP) and weight were observed. Cardiac output (CO), stroke volume (SV), left ventricular end - diastolic volume (LVEDV), left ventricular end - systolic volume (LVESV), left ventricular ejection fraction (LVEF), Peak E, e', and a' decreased, while left ventricular mass index increased. Myocardial work parameters also showed significant changes after LSG, with global longitudinal strain (GLS) and global work efficiency (GWE) increasing and global work index (GWI), global constructive work (GCW), and global wasted work (GWW) decreasing. Significant correlations were observed between the differences in GWW and left ventricular end - diastolic diameter (LVDd), as well as between the differences in GWI and LVEDV. The differences in left ventricular mass and its index were both significantly negatively correlated with the difference in GWW.

**Conclusions:**

LV - PSL can effectively evaluate left ventricular myocardial work in obese patients. Weight loss after LSG can improve left ventricular myocardial work efficiency, and the associated parameter changes are related to cardiac structure, offering new clinical references.

## 1 Introduction

Since 1980, the prevalence of overweight and obesity has increased globally, with nearly one-third of the world's population now affected (1). Obesity is a major risk factor for various chronic diseases, especially cardiovascular diseases (2–4). Adipocytes secrete adipokines, triggering chronic low - grade inflammation. Obesity boosts the heart's workload, activating the mitogen - activated protein kinase (MAPK) pathway in cardiomyocytes and contributing to left ventricular hypertrophy (LVH) (5). Hypertension, common in obesity, raises cardiac afterload. This activates the renin - angiotensin - aldosterone system (RAAS), promoting LVH and fibrosis (6). The adipokine - induced inflammation, along with the hemodynamic stress from obesity and hypertension, promotes cardiac remodeling (6, 7). Obesity also induces metabolic syndrome, compounding the cardiovascular disease risk (6). LSG is one of the commonly used bariatric surgeries, and previous studies have shown that it can improve cardiac structure, such as reducing myocardial hypertrophy and optimizing ventricular chamber size (8, 9). However, its effects on cardiac systolic and diastolic functions remain incompletely understood, which is the basis of this study (7, 10, 11).

The left ventricular strain, based on two - dimensional (2D) speckle - tracking technology, can quantitatively assess left ventricular systolic function of obese patients. However, its major drawback is load dependence. When the left ventricular after-load increases, the strain decreases, impairing the evaluation of myocardial systolic function (12). LV - PSL combines left ventricular global longitudinal strain (GLS) with left ventricular systolic blood pressure based on 2D speckle - tracking technology, so it can accurately evaluate left ventricular myocardial work (13). The aim of this study was to investigate the impact of post-LSG weight loss on LVMW in obese patients, and to explore the clinical value of LV - PSL.

## 2 Materials and methods

### 2.1 Research subjects

A total number of 38 obese patients (BMI ≥ 30 kg/m^2^) hospitalized in Henan Provincial People's Hospital from November 2018 to January 2020 were selected as the preoperative group, including 20 males (53%) and 18 females (47%). The average age of the patients was 32.76 ± 6.10 years. Obesity is defined as a BMI ≥ 30 kg/m^2^ and overweight as a BMI ≥ 25 kg/m^2^ in this study, which is widely used in international research and is also applicable to our study population considering the characteristics of the patients we recruited. During the six - month post - LSG follow - up, seven patients were lost to follow - up. Thirty - one patients who met the BMI criteria of 25.0–29.9 kg/m^2^ were included in the study, with 17 males (55%) and 14 females (45%) and an average age of 33.19 ± 6.40 years. Exclusion criteria included severe liver or renal dysfunction, structural heart disease, secondary obesity, age < 18 years, and refusal to participate. Before enrollment, all patients underwent an electrocardiogram (ECG) examination. Only those with normal QRS complexes, without left bundle - branch block (LBBB) or right bundle - branch block (RBBB), were included in the study. This was to ensure that potential confounding factors associated with abnormal ventricular depolarization and repolarization did not influence the evaluation of myocardial structure and function by echocardiography. All participants underwent a thorough screening for aortic stenosis. This involved a detailed evaluation of the aortic valve morphology using two-dimensional echocardiography and an assessment of the transaortic valve flow velocities by Doppler echocardiography. After a meticulous examination, it was determined that none of the participants had aortic stenosis. The LSG procedures were all performed by the same team. This study was approved by the Ethics Committee of Henan Provincial People's Hospital with a serial No. 2019 and an Ethical Review No. 34, and all subjects signed informed consent forms.

### 2.2 Instruments and methods

#### 2.2.1 Instruments

A GE Vivid E95 ultrasound diagnostic system (GE Vingmed Ultrasound, Horten, Norway), equipped with a M5SC - D heart probe (frequency: 1.5–4.6 MHz, frame rate: 50–80 frames/s) was used. Echo Pac Software (Echo Pac 203 rev 66.4 GE Healthcare, Solna, Sweden) was also utilized.

#### 2.2.2 Image collection

All subjects were placed in the left lateral decubitus position and connected to chest - lead ECG recording devices. Transthoracic echocardiography was performed under calm breathing. Standard echocardiographic parameters such as left ventricular end - systolic diameter (LVDs), left ventricular end - diastolic diameter (LVDd), left ventricular posterior wall thickness (LVPWT), and interventricular septal thickness (IVST), peak early (E) and late (A) diastolic mitral inflow velocities, early and late diastolic mitral annular velocities at the left ventricular lateral wall and ventricular septal annular site and their average values (e') and (a') were measured. Through careful analysis of the Doppler flow patterns and color Doppler images, no significant mitral regurgitation was detected in any of the subjects. LVEDV, LVESV and SV were measured in the left ventricular apical four - chamber and two - chamber views using the biplane Simpson's method. LVEF and CO were calculated based on these measurements. During the image analysis, the left ventricular mass (LV mass) was calculated according to the Devereux formula (LV mass = 0.8 × [1.04 × ((LVDd + IVST + LVPWT)^3^ − LVDd^3^)] + 0.6, with LVDd being the left ventricular end - diastolic diameter, IVST the interventricular septal thickness, and LVPWT the left ventricular posterior wall thickness). Meanwhile, the body surface area (BSA) was calculated using the Du Bois formula (BSA = 0.007184 × height (cm)^0.725^ ×  weight (kg)^0.425^), and then the LV mass index (LVMI) was derived (LV mass index = LV mass/BSA). All measurements were repeated three times by two experienced clinicians to obtain the mean values. Five consecutive cardiac cycle dynamic images from the left ventricular apical four - chamber, three - chamber, and two - chamber views were acquired and stored for offline analysis.

Although it has been suggested that parameters such as E/A, E/e', pulmonary artery systolic pressure (PASP), tricuspid annular plane systolic excursion (TAPSE), and tricuspid valve systolic velocity (TVSa) are suggested to be important in evaluating cardiac function changes related to weight loss, they were not included in our data collection protocol due to design limitations at the time. Future research endeavors in this area should aim to incorporate these measurements to provide a more comprehensive analysis.

#### 2.2.3 Blood pressure measurement

The blood pressure of the participants was measured using a standard mercury sphygmomanometer. Subjects were seated in a comfortable chair with their arms positioned at heart level. After a resting period of 5–10 min, the blood pressure was measured on the brachial artery of the non-dominant arm. Systolic blood pressure was identified as the first Korotkoff sound, and diastolic blood pressure was noted as the fifth Korotkoff sound. Three consecutive measurements were taken at intervals of 2–3 min, and the average of these values was used for further data analysis.

#### 2.2.4 Offline image analysis

**Figure 1:**
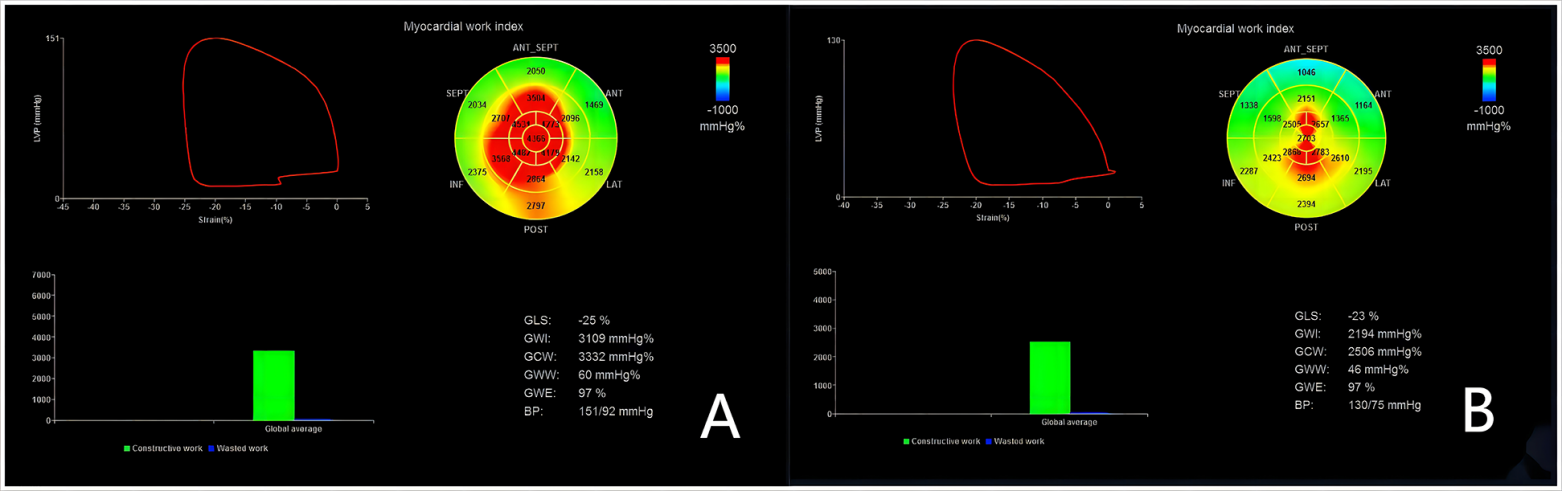
Left ventricular myocardial work measurements with the left ventricular pressure-strain loop (LV-PLS). The left ventricular global and segmental myocardial work are illustrated, with red region indicating high work and blue region indicating low work. **(A)** depicts the LV - PSL of a representative patient preoperatively, and **(B)** shows that of the same patient postoperatively. Note that these do not represent the entire groups but illustrate the general trends and differences in myocardial work before and after sleeve gastrectomy.

The left ventricular myocardial GLS was measured by 2D speckle - tracking with the Echo PAC 203 workstation software. Then, in the BE + TRACE system, when the left ventricular apical four -, three -, and two - chamber views automatically recognized the aortic valve closure time and the blood pressure was input, GCW, GWE, GWI, GWW and the LV - PSL (Figure 1) were obtained. GCW is the total work performed by the myocardium that is needed to achieve LVEF. It is calculated as the sum of the left ventricular shortening work during systole and the elongation work during isovolumic relaxation, and it is expressed in mmHg%. GWW defines the unproductive work for LVEF, which is a combination of lengthening during systole and shortening during isovolumic relaxation. It is calculated as the sum of the lengthening work during systole and the shortening work during isovolumic relaxation, expressed in mmHg%. GWE is the ratio of GCW to the sum of GCW and GWW, indicating the efficiency of myocardial work. Mathematically, GWE = GCW/(GCW + GWW) × 100%. GWI is calculated as the area under the left ventricular pressure - strain loop curve from the mitral valve closure to the mitral valve opening. This curve is generated by integrating the instantaneous pressure and strain values throughout this specific phase of the cardiac cycle. It represents the total work done by the myocardium during this cardiac cycle phase and is measured in mmHg%.

During the study design and data collection process, we did not specifically collect information regarding the presence of metabolic syndrome among the participants. This was due to limited resources for additional testing and time constraints in the study timeline. As a result, we are unable to analyze the potential influence of metabolic syndrome on weight loss in this study.

During the study period, we closely monitored any changes in the patients’ cardiological therapy and the occurrence of cardiovascular events. Patients were regularly interviewed, and their medical records were thoroughly reviewed. In case of any alterations in treatment or the presence of cardiovascular incidents, we analyzed their potential influence on the study outcomes. Appropriate statistical adjustments were made during data analysis to ensure the reliability of the results.

### 2.3 Statistical analysis

Data were analyzed using SPSS 22.0 software. Continuous variables were expressed as mean ± SD. Due to a decrease in the number of patients during follow-up, only patients with complete follow-up data were included in the final analysis (*n* = 31). Categorical variables were expressed as numbers and percentages. The normality of the studied parameters was tested using the Shapiro - Wilk test. For normally distributed data, paired - sample *t*-test was used for comparison; otherwise, the Wilcoxon test was used. To explore the relationships among key parameters, Pearson's correlation coefficient was calculated for variables with approximate normal distribution, such as GWW difference and LVDd difference, GWI difference and LVDd difference before and after LSG, with their significance judged by *P*-values. For variables not meeting normality or potentially having non-linear relationships like BMI difference and LVEF difference before and after LSG, Spearman's rank correlation coefficient was employed. The intra- and inter-class correlation coefficients (ICCs) were calculated to assess the repeatability of the measurements, and inter- and intra-observer repeatability tests were carried out. *P* < 0.05 was considered statistically significant in all tests.

## 3 Results

### 3.1 General clinical data and 2D echocardiographic parameters before and after sleeve gastrectomy

The general clinical data parameters before and after sleeve gastrectomy are presented in Table 1. After LSG, significant changes were observed in several parameters. BMI, DBP and weight decreased significantly. The evaluation of the 2D echocardiographic measurements before and after sleeve gastrectomy are summarized in Tables 2, 3. CO, SV, LVEDV, LVESV, LVEF, Peak E, e', and a' decreased, while LVMI increased. IVST changed from 10.13 ± 1.77 mm before LSG to 10.16 ± 1.57 mm after LSG, with no significant difference (*P* = 0.940). LV mass was 184.76 ± 33.81 g after LSG, compared with 190.04 ± 31.90 g before LSG. Although there was a downward trend, the difference was not significant (*P* > 0.05). No statistically significant differences were found between the pre- and postoperative values of the other parameters (*P* > 0.05).

**Table 1:** Comparison of clinical data parameters before and after sleeve gastrectomy^a^.

Group	Number of cases	Male, *n* (%)	Age (years)	Height (cm)	Heart rate (beats/min)	SBP (mmHg)	DBP (mmHg)	BMI (kg/m^2^)	Weight (kg)
Preoperative group	31	17 (55%)	33.19 ± 6.40	161.51 ± 4.94	75.26 ± 8.43	129.61 ± 8.58	78.07 ± 5.63	35.97 ± 3.76	93.90 ± 10.92
Postoperative group	31	17 (55%)	33.19 ± 6.40	161.51 ± 4.94	74.87 ± 8.64	127.81 ± 7.60	75.32 ± 6.32	27.23 ± 1.41	71.06 ± 5.42
*T-*value/Z-value					1.753	1.544	2.707	12.950	108.703
*P-*value					0.090	0.133	0.011	<0.001	<0.001

^a^ Date are presented as mean ± SD.

A *P*-value < 0.05 was considered to indicate a statistically significant difference.

SBP, systolic blood pressure; DBP, diastolic blood pressure; BMI, body mass index.

**Table 2:** Comparison of conventional echocardiographic parameters before and after sleeve gastrectomy^a^.

Group	LVDd (mm	LVDs (mm)	IVST (mm)	LVPWT (mm)	CO (l/min)	SV (ml)	LV mass (g)	LVMI (g/m^2^)
Preoperative group	50.16 ± 3.41	33.03 ± 2.74	10.13 ± 1.77	10.35 ± 1.31	6.25 ± 0.57	55.61 ± 6.00	190.04 ± 31.90	93.10 ± 17.04
Postoperative group	49.19 ± 3.67	33.00 ± 2.71	10.16 ± 1.57	10.29 ± 1.22	5.69 ± 0.72	51.03 ± 7.86	184.76 ± 33.81	103.89 ± 20.28
*T-*value/Z-value	1.015	1.000	0.006	1.438	3.163	3.152	0.399	5.145
*P-*value	0.318	0.325	0.940	0.161	0.004	0.004	0.530	0.027

^a^ Date are presented as mean ± SD.

A *P*-value < 0.05 was considered to indicate a statistically significant difference.

LVDd, left ventricular end diastole diameter; LVDs, left ventricular end systolic diameter; IVST, interventricular septal thickness; LVPWT, left ventricular posterior wall thickness; CO, cardiac output; SV, stroke volume; LV mass, left ventricular mass; LVMI, left ventricular mass index.

**Table 3:** Comparison of conventional echocardiographic parameters before and after sleeve gastrectomy^a^.

Group	LVEDV (ml)	LVESV (ml)	LVEF (%)	A (m/s)	E (m/s)	e’ (cm/s)	a' (cm/s)
Preoperative group	97.97 ± 4.85	42.35 ± 3.44	71.13 ± 3.29	0.80 ± 0.14	1.13 ± 0.15	0.25 ± 0.04	0.16 ± 0.04
Postoperative group	89.45 ± 6.45	38.42 ± 5.55	67.68 ± 4.70	0.78 ± 0.14	1.04 ± 0.16	0.23 ± 0.04	0.14 ± 0.02
*T-*value/Z-value	7.706	3.365	3.123	0.556	3.540	2.858	2.711
*P-*value	<0.001	0.002	0.004	0.583	0.001	0.008	0.011

^a^ Date are presented as mean ± SD.

A *P*-value < 0.05 was considered to indicate a statistically significant difference.

LVEDV, left ventricular end diastolic volume; LVESV, left ventricular end systolic volume; LVEF, left ventricular ejection fraction; A, peak late diastolic mitral inflow velocity; E, peak early diastolic mitral inflow velocity; e', early diastolic mitral annular velocity at the lateral annular site; a', late diastolic mitral annular velocity at the lateral annular site.

### 3.2 Comparison of GLS and myocardial work parameters before and after sleeve gastrectomy

The results of the evaluation of the myocardial work parameters by PSL before and after sleeve gastrectomy are summarized in Table 4. Myocardial work parameters also showed significant changes, with GLS and GWE increasing and GWI, GCW, and GWW decreasing. All the differences were statistically significant.

**Table 4:** Comparison of GLS and myocardial work parameters before and after sleeve gastrectomy^a^.

Group	GLS (%)	GWI (mmHg%)	GCW (mmHg%)	GWW (mmHg%)	GWE (%)
Preoperative group	−25.19 ± 3.91	2,524.52 ± 86.71	2,624.77 ± 266.76	97.74 ± 6.80	96.38 ± 0.41
Postoperative group	−20.87 ± 4.11	2,300.45 ± 185.12	2,493.19 ± 189.31	66.84 ± 5.92	97.37 ± 0.32
*T-*value/Z-value	−4.209	7.047	2.509	20.484	−13.690
*P-*value	<0.001	<0.001	0.018	<0.001	<0.001

^a^ Date are presented as mean ± SD.

A *P*-value < 0.05 was considered to indicate a statistically significant difference.

GLS, global longitudinal strain; GWI, global work index; GCW, global constructive work; GWW, global wasted work; GWE, global work efficiency.

### 3.3 Repeatability parameters

Thirty patients were selected randomly for assessment of their inter- and intra-observer repeatability. The intra- and inter-observer ICC values for GLS were 0.959 and 0.933, respectively. The intra- and inter-observer ICC values for GWW were 0.981 and 0.941, correspondingly. The intra- and inter-observer ICC values for GCW were 0.967 and 0.923, respectively; for GWI, the intra- and inter-observer ICC values were 0.971 and 0.943, respectively, and for GWE were 0.977 and 0.931, correspondingly. These results showed that the repeatability was good.

### 3.4 Correlation analysis

Table 5 summarizes the correlations between the differences in diverse cardiac parameters and BMI before and after LSG. Most correlations involving BMI difference, such as those with the differences in GWW, GWE, GLS, and GCW, showed no strong linearity (*P* > 0.05). However, potential trends might exist for further study. The correlation between the differences in BMI and LVEF demonstrated a weak negative relationship in Spearman's analysis (*ρ* = −0.148, *P* = 0.428). Tables 6, 7 summarize the correlations between the differences in myocardial work parameters and LV mass as well as LVMI, both before and after LSG. Notably, there existed a significant negative correlation between the differences in both LV mass and LVMI and GWW. Significant correlations were observed between the differences in GWW with LVDd (*r* = −0.405, *P* = 0.026), and also between the differences in GWI with LVEDV (*r* = 0.529, *P* = 0.002).

**Table 5:** Correlations between the differences in diverse cardiac parameters and BMI before and after sleeve gastrectomy.

Group	*r-*value/*ρ*-value	*P-*value
*Δ*GCW	0.101	0.589
*Δ*GWI	−0.315	0.085
*Δ*GWW	0.181	0.331
*Δ*GWE	−0.097	0.603
*Δ*GLS	−0.049	0.794
*Δ*LVEF	−0.148	0.428

A *P*-value < 0.05 was considered to indicate a statistically significant difference.

*Δ*GCW represents the difference in global constructive work before and after gastrectomy; *Δ*GWI for the difference in global work index before and after gastrectomy; *Δ*GWW for the difference in global wasted work before and after gastrectomy; *Δ*GWE for the difference in global work efficiency before and after gastrectomy; *Δ*GLS for the difference in global longitudinal strain before and after gastrectomy; *Δ*LVEF for the difference in left ventricular ejection fraction before and after gastrectomy; BMI: body mass index.

**Table 6:** Correlations between the differences in myocardial work parameters and LV mass before and after sleeve gastrectomy.

Group	*r-*value/*ρ*-value	*P-*value
*Δ*GCW	−0.116	0.535
*Δ*GWI	−0.069	0.712
*Δ*GWW	−0.403	0.024
*Δ*GWE	−0.020	0.916
*Δ*GLS	−0.206	0.265

A *P*-value < 0.05 was considered to indicate a statistically significant difference.

*Δ*GCW represents the difference in global constructive work before and after gastrectomy; *Δ*GWI for the difference in global work index before and after gastrectomy; *Δ*GWW for the difference in global wasted work before and after gastrectomy; *Δ*GWE for the difference in global work efficiency before and after gastrectomy; *Δ*GLS for the difference in global longitudinal strain before and after gastrectomy; LV mass: left ventricular mass.

**Table 7:** Correlations between the differences in myocardial work parameters and LVMI before and after sleeve gastrectomy.

Group	*r-*value/*ρ*-value	*P-*value
*Δ*GCW	−0.082	0.662
*Δ*GWI	−0.083	0.655
*Δ*GWW	−0.438	0.014
*Δ*GWE	0.024	0.899
*Δ*GLS	−0.115	0.540

A *P*-value < 0.05 was considered to indicate a statistically significant difference.

*Δ*GCW represents the difference in global constructive work before and after gastrectomy; *Δ*GWI for the difference in global work index before and after gastrectomy; *Δ*GWW for the difference in global wasted work before and after gastrectomy; *Δ*GWE for the difference in global work efficiency before and after gastrectomy; *Δ*GLS for the difference in global longitudinal strain before and after gastrectomy; LVMI: left ventricular mass index.

## 4 Discussion

Obesity is a well-established global health concern and a notably significant independent risk factor for cardiovascular diseases (14). Notably, previous studies have precisely shown that for every 1 kg/m^2^ increment in BMI, the risk of heart failure experiences an average elevation of 5% in obese men and 7% in obese women, while the risk of atrial fibrillation witnesses an approximate 4% increase (15, 16). The pathophysiological mechanisms underlying this association are multifactorial. Adipocytes in obese individuals secrete various adipokines, which disrupt normal metabolic processes and trigger chronic low-grade inflammation. This inflammatory state, in turn, promotes cardiac remodeling, characterized by ventricular hypertrophy and fibrosis, and contributes to the development of metabolic syndrome, which encompasses abdominal obesity, glucose intolerance, lipid disorders, and hypertension, all of which synergistically increase the risk of CVDs (6, 14). This study investigated the effects of weight loss after LSG on left ventricular myocardial work in obese patients. The findings have provided valuable insights into the cardiac adaptations associated with weight loss.

The results demonstrated significant changes in multiple parameters after LSG. We observed that the majority of patients in the preoperative group achieved significant weight loss and transitioned to the overweight category six months after LSG. The notable reductions in BMI, DBP and weight suggest a positive impact on the cardiovascular risk profile. Concomitantly, the decreases in CO, SV, LVEDV, LVESV, and LVEF indicate a reduction in cardiac workload. The reduction in LVEDV and LVESV is indicative of a diminished preload on the heart. This can be primarily attributed to the improvement in metabolism and the reduction in systemic blood volume following weight loss. This suggests that the heart is progressing towards a more physiological state, which is likely a result of the alleviation of the excessive burden imposed by obesity on the cardiovascular system. Such changes might be attributed to the improved metabolic status and decreased systemic blood volume following weight loss, as well as the potential reversal of the compensatory mechanisms that were initially activated in response to the obese state. This could involve the regression of left ventricular myocardial remodeling, which was previously induced by the increased demand for peripheral blood circulation in obese patients. This is consistent with previous research indicating that weight loss can alleviate the excessive burden placed on the heart by obesity (14, 17). In obese patients, weight gain elevates the demand for peripheral blood circulation, triggering compensatory left ventricular myocardial remodeling. Despite low peripheral resistance, they have high CO, which may cause left ventricular growth and hypertrophy (18). LSG surgery reduces BMI and CO, facilitating a reversible repair of the cardiac structure before surgery and restoring left ventricular function partly, aligning with prior studies (19–23).

Among the cardiac structural parameters, there was no statistically significant change in IVST after LSG, which is not entirely consistent with previous reports (8, 10, 19, 22). The results of this study indicate that in obese patients, when their body weight decreases, the cardiac load changes to some extent. However, the heart may maintain the overall functional balance through other means, so that the IVST does not need to change significantly. It could also be caused by human factors, equipment differences, measurement angles and other issues during the measurement process. And the exact underlying mechanism remains elusive and necessitates further investigation.

In addition to the changes in traditional echocardiographic parameters, the alterations in LV mass and LVMI also provide important insights into the cardiac remodeling process after LSG. In the obese state, the heart endures a high load for a long time, causing an increase in LV mass to maintain normal cardiac function. After LSG, LV mass showed a downward trend, yet the difference was not significant. This indicates that the surgery may have improved certain pathological conditions of the heart to some extent, reducing the pressure or volume load on the left ventricle. As a result, the compensatory hypertrophy of cardiomyocytes has been alleviated, leading to a downward trend in left ventricular mass (24, 25). From the myocardial cell metabolism perspective, in obesity, high cardiac load prompts myocardial cells to increase protein synthesis by signaling pathways such as the mammalian target of rapamycin (mTOR) pathway, leading to hypertrophy and increased LV mass. After LSG, although the cardiac load and metabolic demands drop, the mTOR pathway's inertia keeps protein synthesis from decreasing rapidly, resulting in a non - significant LV mass decrease. Regarding cardiac remodeling, obesity causes myocardial cell hypertrophy and interstitial fibrosis, increasing LV mass. After LSG, although the cardiac load is reduced and reverse remodeling begins, the improvement of interstitial fibrosis is a slow process. The degradation and remodeling of the extracellular matrix take a long time, which restricts the rapid reduction of LV mass, thus presenting a non - significant decrease trend. The significant increase in the LVMI may reflect that the reduction in BSA is greater than that in LV mass, and it also indicates that there are still certain adaptive changes in the cardiac structure, which is inconsistent with the results of previous studies (24, 25). Our findings may be related to the characteristics of our study population, such as the relatively young age and specific body composition of our patients. Functionally, with a reduced BSA, the heart may enhance contractility or adjust the heart rate to maintain cardiac output. Structurally, myocardial cells may change shape and arrangement, and the relationship between cardiac chambers, myocardial thickness, and BSA is being re - adjusted for more efficient post - surgery function. These findings regarding LV mass and LVMI further support the overall conclusion that LSG - induced weight loss has a complex impact on cardiac structure and function.

LVMW assessment helps detect early myocardial function changes in obese patients, enabling timely clinical intervention and improving their quality of life (26). GLS can assess cardiac function earlier than LVEF but is load-dependent (27). Noninvasive LV-PSL, combining left ventricular deformation and pressure, overcomes this, sensitively and quantitatively assessing myocardial work and oxygen consumption (13, 18). In the study, our results confirmed a significant improvement in the myocardial work parameters in obese patients who achieved a significant weight loss as a result of bariatric surgery. The observed increase in GLS and GWE, along with the decreases in GWI, GCW, and GWW, suggest an improvement in myocardial work efficiency (28). In comparison with the research letter penned by Cecha P et al. (17), our study also showed significant weight loss and changes in GLS, GWE etc. after LSG. But sample characteristics differed. Ours had a mean age of 33.19 ± 6.40 years, 55% male, the research letter penned by Cecha P et al. had different age, gender, and comorbidities. These caused variations in myocardial work parameter changes and correlations. In our study, the correlations between the differences in myocardial work parameters and BMI before and after LSG showed no strong linearity. The research letter had different data. This comparison reveals the complexity of LSG-induced myocardial work changes, offering a broader view for future studies. In the patients without abnormal myocardial changes, GWE was higher due to lower GWW. Conversely, the decrease in GWE indicates that left ventricular myocardial synchronous systolic function, which is the key factor to maintaining normal cardiac function, has been impaired (26, 29). From the perspective of myocardial mechanics, the aforementioned results indicate that weight loss and BMI reduction after LSG surgery could diminish myocardial oxygen consumption and ineffective myocardial work, repair myocardial remodeling, and improve left ventricular myocardial synchronous systolic function, thereby potentially prolonging cardiac longevity. The findings of this study confirm that myocardial work function in obese patients can be improved by reducing their weight, thereby reversing some of the earlier developed pathological changes.

Cardiovascular imaging studies have shown the adverse effects of obesity on the left ventricular structure and function (30, 31). To enhance our understanding of the relationships between myocardial work and cardiac structure/function, we conducted correlation analyses. We found significant correlations between the differences in GWW and LVDd, as well as between the differences in GWI and LVEDV, holding great pathophysiological significance. In the obese state, the increase in adipose tissue leads to an expansion of blood volume, increasing the preload on the heart, which in turn dilates LVDd and LVEDV. This means that the heart has to pump a larger volume of blood each time, and the myocardium needs to do more work to maintain cardiac output, as reflected by the elevation of myocardial work parameters such as GWI and GCW. After LSG, weight loss leads to improved metabolism and a reduction in blood volume, causing LVDd and LVEDV to decrease. The decline in GWI and GCW at this time indicates that due to the reduced preload, the heart no longer needs to perform excessive work to maintain normal cardiac output, which is an indication of left ventricular unloading. For example, a decrease in LVEDV reduces the volume of blood that the heart needs to pump during systole, alleviating the burden on myocardial contraction and reducing myocardial oxygen consumption, thereby improving the heart's work efficiency. This change helps to relieve the stress on the heart, reducing the risk of myocardial hypertrophy and cardiac remodeling, and has a positive impact on the improvement of cardiac function. These findings suggest that alterations in myocardial work are intricately linked to changes in left ventricular geometry and function, providing a basis for further elucidating the mechanisms underlying these changes (32–37). The relationship among GWW, LV mass and LVMI is complex. Theoretically, a reduction in GWW and LV mass should exhibit a positive correlation, as both reflect improvements in cardiac efficiency and structure following weight loss, as supported by previous studies (17, 24). However, our study found a negative correlation, which can be attributed to the following factors: GWW reflects immediate myocardial work efficiency, which improves rapidly with postoperative load reduction. After LSG, weight loss quickly reduces cardiac load, leading to a significant drop in GWW and an immediate improvement in myocardial efficiency. Conversely, the reversal of LVH and myocardial remodeling is a slow process that takes months to years. During the six - month post - LSG follow - up, although the cardiac load is reduced, LV mass may not decrease significantly because of compensatory mechanisms such as residual hypertrophy or fibrosis. Thus, the rapid improvement in GWW and the slow change or even increase (due to BSA reduction) in LV mass LVMI result in a negative correlation. LVMI is calculated as LV mass divided by BSA. After LSG, a significant reduction in BSA may cause LVMI to increase even if LV mass remains relatively stable. This mathematical relationship contributes to a negative correlation between the differences in GWW (reduced) and LVMI (increased). Functional improvements like reduced GWW may occur earlier than structural adaptations in LV mass. During the six - month post - LSG follow - up, this temporal discrepancy can lead to a negative correlation between the differences in GWW and LV mass. Patient - to - patient variability also plays a role. Some patients show a positive correlation between changes in GWW and LV mass, while others show a negative one. These individual differences affect the overall result and contribute to the observed negative correlation in our study. Future long - term, large - scale studies are needed to clarify if the correlation turns positive over time and to better understand the cardiac changes after weight - loss surgery.

Clinically, our findings offer valuable guidance for the management of obese patients undergoing sleeve gastrectomy. The observed changes in myocardial work parameters suggest that physicians should closely monitor patients' cardiac function not only through traditional echocardiographic measures such as LVEF but also by incorporating LV-PSL-derived parameters. For patients with significant changes in myocardial work efficiency, individualized cardiac rehabilitation programs could be designed. These programs may include tailored exercise regimens and dietary advice to further optimize cardiac function and reduce the risk of future cardiovascular events. Moreover, the identified correlations between myocardial work parameters and other cardiac structural and functional parameters can assist in risk stratification. Patients with specific patterns of changes in these parameters could be identified as being at higher risk, warranting more intensive follow-up and potentially earlier intervention. This personalized approach has the potential to improve the long-term outcomes of obese patients after bariatric surgery and enhance their overall quality of life.

This study has several limitations. It is a single-center study with a small sample size, only evaluating the short-term effects of LSG based on six-month follow - up data, lacking long-term outcome comparison. Brachial artery pressure was used instead of left ventricular pressure with some deviations. Also, only global myocardial work was assessed without exploring regional work by segments.

Regarding obesity phenotypes, Preda et al. identified metabolically unhealthy normal weight (MUNW), metabolically healthy overweight/obese (MHO), metabolically unhealthy overweight/obese (MUO), and sarcopenic obesity (SO) (14). MUO has metabolic syndrome features and high cardiovascular risk; MHO has relatively lower risk but may develop problems over time; MUNW has normal weight but abnormal fat and metabolism with increased risk; SO combines muscle loss and obesity, which further exacerbates the cardiovascular burden. Our study didn't classify patients by phenotypes, but they might have influenced results. Future studies should classify phenotypes to clarify the LSG-weight loss-cardiac work relationship.

Parameters like E/A, E/e', PASP, TAPSE, and TVSa were not included due to design and resource limitations. Future research should add them for more insights into LSG's cardiac effects. Another notable limitation is the absence of data on metabolic syndrome. Metabolic syndrome is known to potentially impact weight loss outcomes (38), and without this information, we cannot fully assess its role in the context of our study. Future studies should consider incorporating the assessment of metabolic syndrome to provide a more comprehensive understanding of the factors influencing weight loss after sleeve gastrectomy and its relationship with cardiac function.

In summary, LV - PSL is a valuable tool for evaluating myocardial work in obese patients after LSG. Weight loss after LSG improves myocardial work efficiency, and the parameter changes are related to cardiac structure and function. Nevertheless, larger - scale, multi - center studies with longer follow - up are required for further verification.

## Data Availability

The original contributions presented in the study are included in the article/Supplementary Material, further inquiries can be directed to the corresponding author.
